# Revisiting Mechanism of Silicon Degradation in Li-Ion Batteries: Effect of Delithiation Examined by Microscopy Combined with ReaxFF

**DOI:** 10.1021/acs.jpclett.4c03620

**Published:** 2025-02-21

**Authors:** Carl Erik L. Foss, Mahdi K. Talkhoncheh, Asbjørn Ulvestad, Hanne F. Andersen, Per Erik Vullum, Nils Peter Wagner, Kenneth Friestad, Alexey Y. Koposov, Adri van Duin, Jan Petter Mæhlen

**Affiliations:** † Department of Battery Technology, Institute for Energy Technology, P.O. Box 40, NO-2027 Kjeller, Norway; ‡ Department of Chemical Engineering, Pennsylvania State University, University Park, Pennsylvania 16802, United States; § Centre for Material Science and Nanotechnology, Department of Chemistry, University of Oslo, P.O. Box 1033, Blindern, 0371 Oslo, Norway; ∥ Elkem, P.O. Box 8040, Vaagsbygd, NO-4675 Kristiansand, Norway; ⊥ SINTEF Industry, P.O. Box 4760, NO-7465 Trondheim, Norway

## Abstract

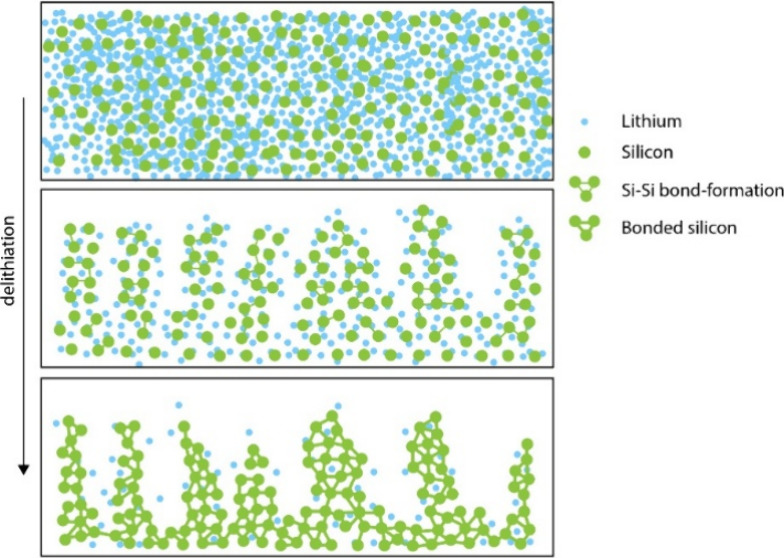

For the past decade, silicon (Si) as a material for negative electrodes of Li-ion batteries has been considered among the most promising candidates for replacing commonly used graphite. However, Si-based electrodes suffer from severe degradation, which depends on the type of Si materials used. Generally, the degradation of Si is mainly viewed in terms of particle fracturing during lithiation accompanied by constant growth of the solid electrolyte interphase (SEI). At the same time, the reversed process, delithiation, has received little attention. The present work demonstrates the morphological changes of the Si components of electrodes occurring during electrochemical cycling through electron microscopy analyses. These changes are rationalized through the migration of Si, resulting in the formation of Si dendrites embedded in SEI. With the assistance of ReaxFF modeling, we demonstrate that the delithiation predominantly drives this process. The present study reveals that fracturing of Si particles is not the only cause for degradation, as the Si surfaces dramatically change after prolonged cycling, resulting in the formation of Si dendrites.

Silicon (Si) is considered to be a promising anode material for Li-ion batteries (LIBs), due to its high capacity (∼10 times higher than that of graphite) and relative availability.^1,2^ However, the implementation of Si-based anodes with high amounts of Si still faces significant challenges.^3^ Rapid structural degradation of the Si-based anodes, resulting in fast capacity fading and an increase in the cell’s internal resistance, is the primary reason for the slow implementation of this otherwise-promising material in commercial LIBs.^4^ The primary cause of degradation originates from structural and morphological changes of the Si-based anode due to high Li storage capacity of Si (theoretical capacity of 3579 mAh/g).^5−7^ As a result, the amount of Si in LIB anodes is typically limited to a few percent to strike a balance between harnessing the benefits of its high capacity and mitigating the degradation issues.^3,8^

The design, improvement, and stabilization of Si-based anodes require a detailed understanding of the degradation mechanisms to implement mitigation tactics effectively. The predominant opinion in the literature regarding the degradation mechanisms of Si-based anodes typically highlights fracturing and cracking of Si particles during lithiation as the primary destructive process.^9^ While the problems associated with fracturing could be somewhat mitigated through the introduction of nanostructured materials,^10^ the large volumetric changes of Si during electrochemical cycling lead to a continuous formation of solid electrolyte interphase (SEI) consuming the electrolyte components. While these studies of nanosized Si can provide valuable information about fundamental processes during degradation,^11^ they are not necessarily applicable to realistic commercial anode systems. Due to the low inherent packing density, the overall volumetric energy densities are much lower. Also, the initially high surface-to-volume ratio of nanoparticles leads to a larger fraction of the natural oxide on the surface, an increase in SEI formation, and a low first-cycle Coulombic efficiency.^12^ In addition, the morphological changes occurring in electrodes with micrometer-sized particles differ from those observed in model systems.^13^ For these reasons, studies of Si with particle sizes up to several micrometers are more applicable in a realistic electrode configuration.

Most studies examining the degradation of Si-based anodes focus on the lithiation process and the stresses induced during cycling.^14−17^ Si anodes in LIBs undergo complex structural changes during lithiation and delithiation processes. Crystalline Si typically becomes amorphous during the initial lithium insertion/formation cycle, with a new crystalline phase (Li_15_Si_4_) forming at highly lithiated samples (typically below 50 mV vs Li/Li^+^), resulting in decreased performance.^18^ Molecular dynamics simulations showed that lithiation in amorphous Si is less inhibited kinetically and produces less localized stress, compared to crystalline Si.^19^ The study directly comparing crystalline and amorphous Si of same size and morphology demonstrated better electrochemical performance of amorphous Si anodes than their crystalline analogues.^20^ In addition, a mechanistic study of the complex phase transformation between crystalline and amorphous phases during (de)lithiation of Si anodes showed that observed voltage hysteresis between lithiation and delithiation mainly originates from the transformation between the c-Li_15−δ_Si_4_ and a-Li_15−δ_Si_4_ phases.^21^ Investigation of changes in short-range order during the first cycles of Si anodes with ex-situ Li nuclear magnetic resonance (NMR) combined with pair distribution function (PDF) linked the hysteresis in the electrochemical profile during the initial cycles to the formation of distinct amorphous lithiated silicide structures, forming small clusters, which is consequently broken up in the following cycles to isolated Si.^22^

Only a few publications address the relationship between the delithiation process and Si degradation.^23,24^ While a series of post-mortem microscopy analyses performed at different stages of nanostructured Si cycling shed some light toward understanding the importance of the delithiation process for nanostructured Si,^24^ there remains a need for similar studies on micrometer-sized Si particles. The transition from nanoscale to microscale particles of active materials can provide several beneficial properties in terms of improved gravimetric and volumetric energy density,^25^ and minimizing the first cycle losses.^26^

In the present work, we have performed a sequence of microscopy studies of anodes based on industrially produced Si (a material produced in large commercial quantities) to elucidate the effect of delithiation on the structural and morphological changes occurring in Si-based anodes with micron-sized particles during electrochemical cycling. We have combined our experimental work with reactive force-field (ReaxFF) molecular dynamics simulations to gainatomistic insight into the degradation processes. ReaxFF interatomic potentials bridge the gap between quantum mechanics and empirical interatomic potentials and can be applied to a broad range of systems.^27^ A similar approach was used to investigate Li insertion into c-SiNWs,^17^ and recently showed to provide atomistic insights into understanding materials with conversion–alloying mechanisms.^28^ This combination of techniques highlights the effect of delithiation on the degradation of Si-based anodes.

The properties of micrometer-sized Si from commercial sources used in the present work were described in our previous publication.^29^ The galvanostatic cycling of microscale Si is shown in Figure 1a. The cells have a relatively high first-cycle Coulombic efficiency for Si at 91.6%. However, after the initial two formation cycles, the capacity of the electrode stays well below the theoretical capacity for Si. The capacity continues to decay until a slight recovery is observed (typically after ∼15 cycles). Subsequent cycles show a reasonably stable plateau for ∼50 cycles, before dropping significantly toward cell failure. This performance has previously been reported and is common for micrometer-sized Si particles.^29^ The preliminary galvanostatic cycling allowed us to assess the key points necessary for further characterization, where the selection of the characterization points was based on anticipated changes of the material, relevant for the analysis of degradation (dotted vertical lines in Figure 1a).

**Figure 1:**
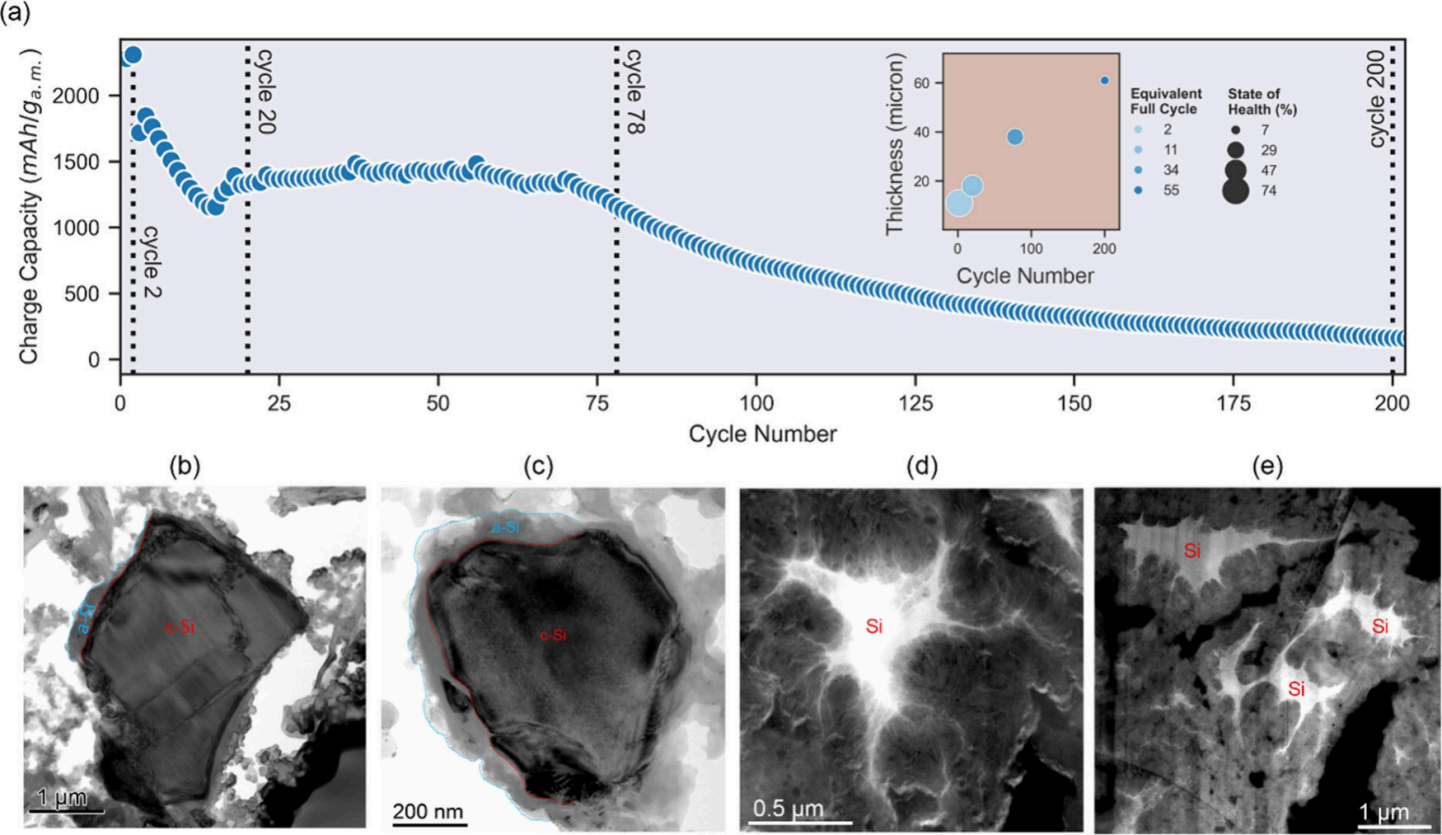
(a) Charge capacity vs cycling for the cell used to estimate the appropriate characterization points (dotted lines indicate where on the capacity plot the post-mortem cells were stopped), inset shows electrode thickness (from FIB-SEM) as a function of cycles, and the EFC and SoH reached for the four cells before being stopped and examined by TEM. Bright-field TEM images after (b) 2 and (c) 20 cycles, and high-angle annular dark-field scanning transmission electron microscopy (HAADF-STEM) images after (d) 78 and (e) 200 cycles. In panels (b) and (c), the blue dashed line shows the surface of the Si particles, while the red dashed line shows the interface between the amorphous shell and the crystalline core. In panels (d) and (e), Si shows bright contrast, while SEI and other carbon-based materials have dark contrast.

Cross sections of the cycled electrodes were analyzed by TEM to visualize the changes in the particle morphology. Figure 1 shows micrographs of micron-sized Si after (b) 2, (c) 20, (d) 78, and (e) 200 charge/discharge cycles. After 2 and 20 cycles, many of the Si particles were observed to have a core–shell structure, with the core comprised of crystalline Si and the amorphous Si shell. The presence of the crystalline core indicates that Si has not yet been fully lithiated during the cycling process and has remained as a passive material, in agreement with the lower capacity observed in the galvanostatic cycling experiments. Through further cycling, the amorphous shell grows and gradually consumes the crystalline core, and, after 78 cycles, only minor solid bodies are present (see Figures S1 and S2 for more-detailed characterization of the structural changes of crystalline/amorphous Si). It should be noted that during the experiments when the electrodes were cycled with limited capacity (1200 mAh/g), the traces of crystalline Si cores could still be found, even after 80 cycles (Figure S3). High-angle annular dark-field scanning transmission electron microscopy (HAADF-STEM) images from electrodes cycled 2, 20, and 100 times are added to the Supporting Information (Figure S4) for comparison.

The TEM analysis revealed a substantial change in the morphology of Si particles between cycles 20 and 78, transforming from relatively smooth surface to extremely rough. A nanoscopic, mossy network of threads and branches of Si has formed, resulting in a very high surface area for each Si particle. These changes in particleś morphology continue to occur between 78 and 200 cycles, and many locations in the electrode also showed significant electrochemical sintering of Si particles where parts of two or several Si particles have fused together, forming bridges between Si particles that appear to have been separated earlier in the cycling process.

As a result of the SEI buildup and degradation, the electrode thickness is expected to increase,^30,31^ and an almost linear thickness increase is observed as a function of cycle number, as shown in the inset of Figure 1a and the cross-section SEM images in Figure 2. After 78 cycles, the thickness was found to be more than 3 times compared to the initial, indicating that most of the electrode volume consists of multiple SEI phases rather than active materials (for detailed comparison of the surface and cross-section SEM of pristine vs after 78 cycles, see Figure S5). Additionally, the porosity decreases as a function of cycling, as evidenced by SEM images (Figure 2). After 200 cycles, large dense regions with a mean size of ∼10–15 μm are separated by larger fractures. This results in longer diffusion pathways for Li ions to pass through the SEI phases, resulting in a buildup of local overpotentials as a function of cycling.

**Figure 2:**
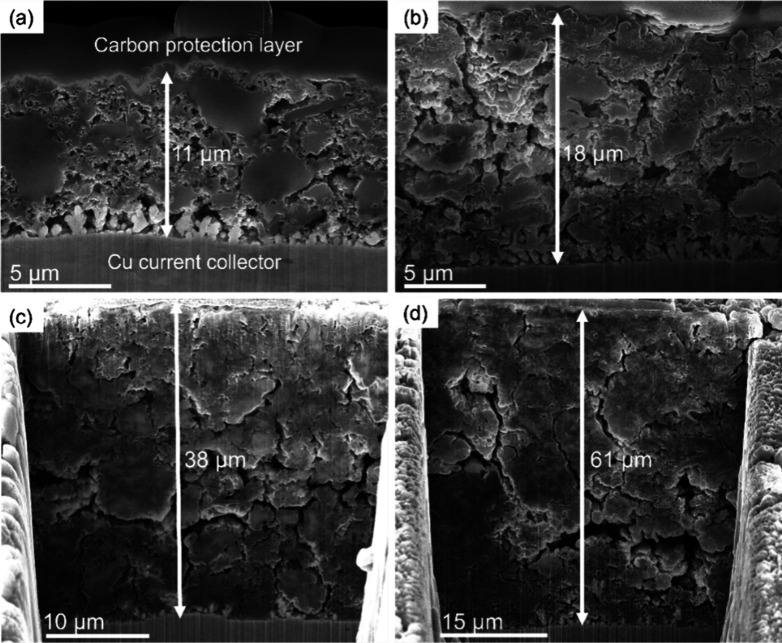
Cross-sectional SEM images of delithiated electrodes after (a) 2, (b) 20, (c) 78, and (d) 200 cycles. Notice that the scale bar is different on all images.

To further examine the chemical evolution of the electrode materials mapping the elements wasn conducted. The element maps of a delithiated electrode after 200 cycles are shown in Figure 3. The Si, O, C, and Li maps were generated from electron energy loss spectroscopy (EELS) data, while the F and P maps were taken from the simultaneously recorded EDS. The cross-sectional SEM imaging already demonstrated the predominant fraction of SEI in the cycled electrode (Figure 2), while the spectroscopy data and element maps in Figure 3 illustrated that O, C, and Li are the dominating elements in the SEI phases. Additionally, significant amounts of F and minor amounts of P were found in the SEI originating from the electrolyte decomposition.

**Figure 3:**
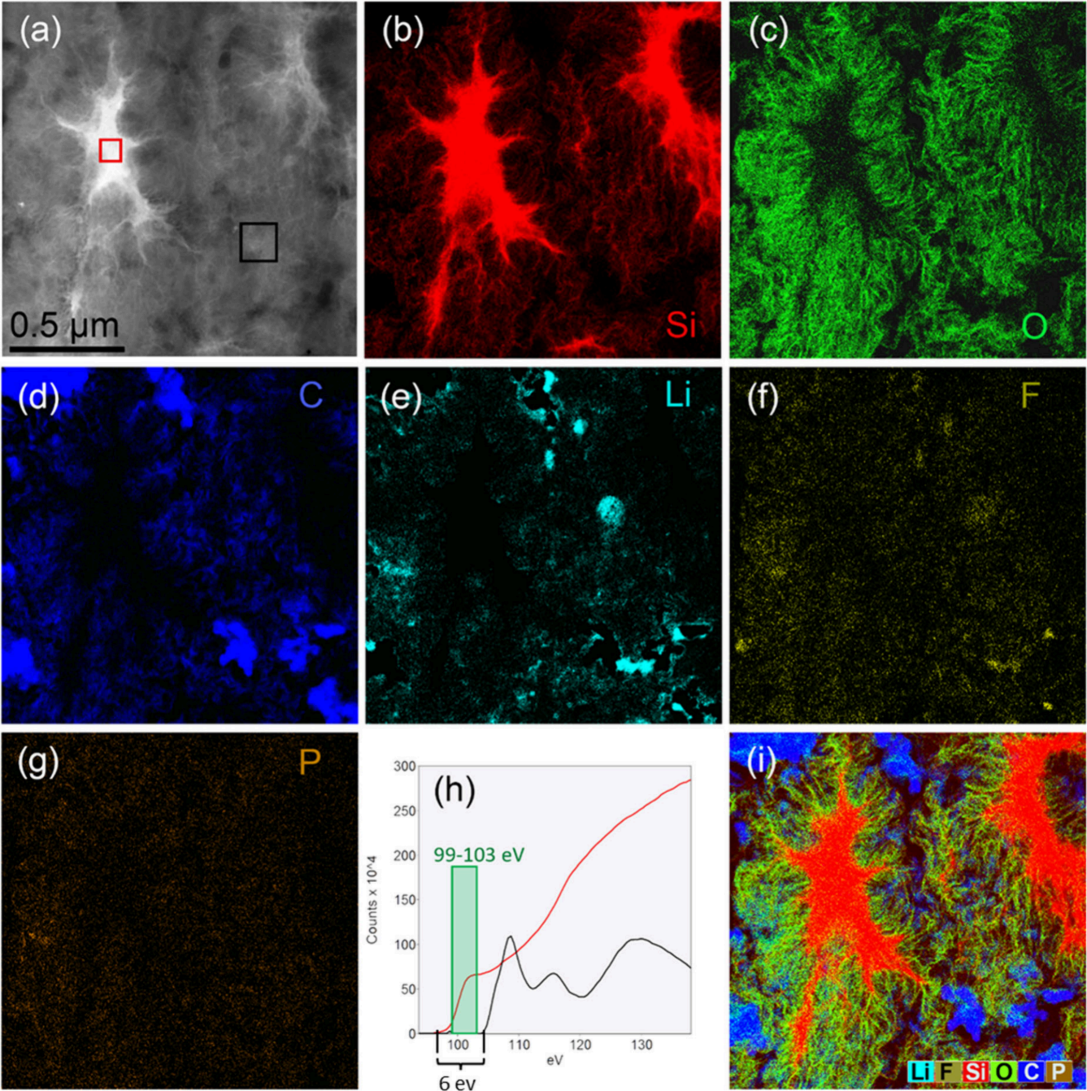
Element mapping by simultaneous electron energy loss spectroscopy (EELS) and X-ray energy dispersive (EDS) spectroscopy. The mapped region is from a delithiated electrode after 200 cycles. (a) High-angle annular dark field scanning transmission electron microscopy image. Panels (b)-(g) show element maps (Si (panel (b)), O (panel (c)), C (panel (d)), Li (panel (e)), F (panel (f)), and P (panel (g))). In panel (h), the Si 2p EEL edge is shown from two regions in the map, marked by red and black squares in the image shown in panel (a). The red line is from a region where Si is amorphous and zerovalent. The black line shows the Si L_2,3_ edge from a region with primarily SEI phases but with a lot of nanoscopic Si embedded inside the SEI. The Si map in image shown in panel (i) is made with the Si signal that falls inside the energy loss window 99–103 eV, as indicated by the green rectangle in panel (h). The false color in the images identifies the different electrode constituents: Si(red), graphite/C (blue), O(green), F(yellow), P(brown), and Li(turquoise).

Further examination of Si mapping revealed two types of Si in the electrodes: Si corresponding to the active particles, and a significant fraction of Si is dispersed as nanoscopic, thread-like structures embedded in the SEI. In Figure 3h, the Si L_2,3_ electron energy loss (EEL) edge from these thread-like structures (black curve) is compared to the Si L_2,3_ edge from a region with pure, amorphous Si (red curve). Comparison of the Si L_2,3_ EEL spectra demonstrate a chemical shift of 6 eV between the solid, amorphous Si particle and Si embedded in the SEI. This increase in the ionization energy of 2p electrons corresponds to a change in the oxidation state of Si from 0 in the Si particles to +4 in Si embedded in the SEI, most likely in the form of lithium silicate.

To confirm that, another Si map was created with electrons that have lost energy only inside the range of 99–103 eV (shown in Figure 3i). Highly oxidized Si, i.e., Si atoms with oxidation states above +2, do not contribute to this energy loss region, and the map Figure 3i shows only Si atoms with oxidation states below +2. Most of the Si present in thread-like structures embedded in the SEI gives no signal to this map. Hence, this Si is no longer active during the cycling but rather likely contributes to the increased internal resistance observed in Figure S6. The Si particles also have a thick (up to several tens of nanometers) surface layer of oxidized Si surrounding them. This oxidized Si surface layer increased in thickness as a function of cycling (shown as comparison of Si vs “pure-Si” on Figure S7). A precise determination of the thickness of this layer is challenging due to the surface morphology of the Si particles combined with the two-dimensional (2D) projections in the spectroscopy maps. However, the surface layer of the Si particles (typically ∼20 nm thick) consistently shows a chemical shift toward a higher oxidation state.

From the data shown above, it becomes evident that the structural changes of Si lead to significant changes in the surface-to-volume ratio and the surface area of Si particles. This phenomenon promotes the further continuous growth of these Si in particle morphological clusters.^32^ While the formation of the remote islands of oxidized Si can be explained by a classical degradation model, which relies on particle fracturing through cycling, the substantial change in morphology of the main Si particles cannot be rationalized through this mechanism. Such tremendous changes could be explained by nonuniform delithiation when particles decrease in size during Li-ionextraction. The localized rates of Li-ion extraction should depend not only on the cycling rates but also particle shapes—higher deviation from spherical shapes will result in a larger distribution of localized Li-ion flows, similar to the Li dendrites when Li metal is used as an electrode. As Si particles used in the present work are nonspherical, it is reasonable to anticipate that lithiated particles will also be nonspherical. Thus, the extraction of Li-ions will promote surface nonuniformity, ultimately enhancing the surface area of Si particles. This process will be continuous and essentially self-promoting, which will result in the formation of observed dendrites. That observation suggests that the delithiation process is important for rationalizing the degradation of Si-based electrodes.

To rationalize this experimental observation and confirm this hypothesis, we have performed reactive force filed (ReaxFF) molecular dynamics simulations. The ReaxFF simulations allow us to elucidate the (de)lithiation mechanism which involves Si–Si bond breakage and subsequent bond formation due to the migration of Si atoms resulting from Li diffusion. A continuous algorithm was developed to capture the effect of different delithiation rates (for details, see the Computational Methods section in the SI and Figures S8 and S9). Specifically, the slabs of Si were lithiated to Li_3.75_Si and then subjected to delithiation similar to methodologies applied in the previous publication.^28^ Delithiation was performed using two different rates of Li-ion removal to understand how the cycling rate can influence the local atomic (re)arrangement. In addition, we have also examined four stages of delithiation were 1500, 3000, 4500, and 6000 Li ions were removed. The latter corresponds to almost complete delithiation of the Li_*x*_Si slab. Figure 4 demonstrates the changes on the Si surface when different delithiation conditions were applied (it should be noted that the original Si surface was flat prior to lithiation). Noteworthy, severe disturbance of the Si surface was observed for all studied cases, but the a-Li_3.75_Si clusters responded differently, depending on the Li removal rate. Slow removal (delithiation) of 6000 Li ions (Figure 4h) seems to favor the formation of more dense Si clusters closer to the initial surface upon full removal. In contrast, faster Li-ion removal leads to highly porous and scattered clusters of Si (Figure 4d). The differences between fast and slow removal are less clear (as both seem rather porous and scattered) for the removal of 1500 and 3000 Li ions (see Figures 4a and 4b and Figures 4e and 4f); however, the delithiation process resulted in substantial migration of Si atoms from their places in the original Si cluster in all studied cases.

**Figure 4:**
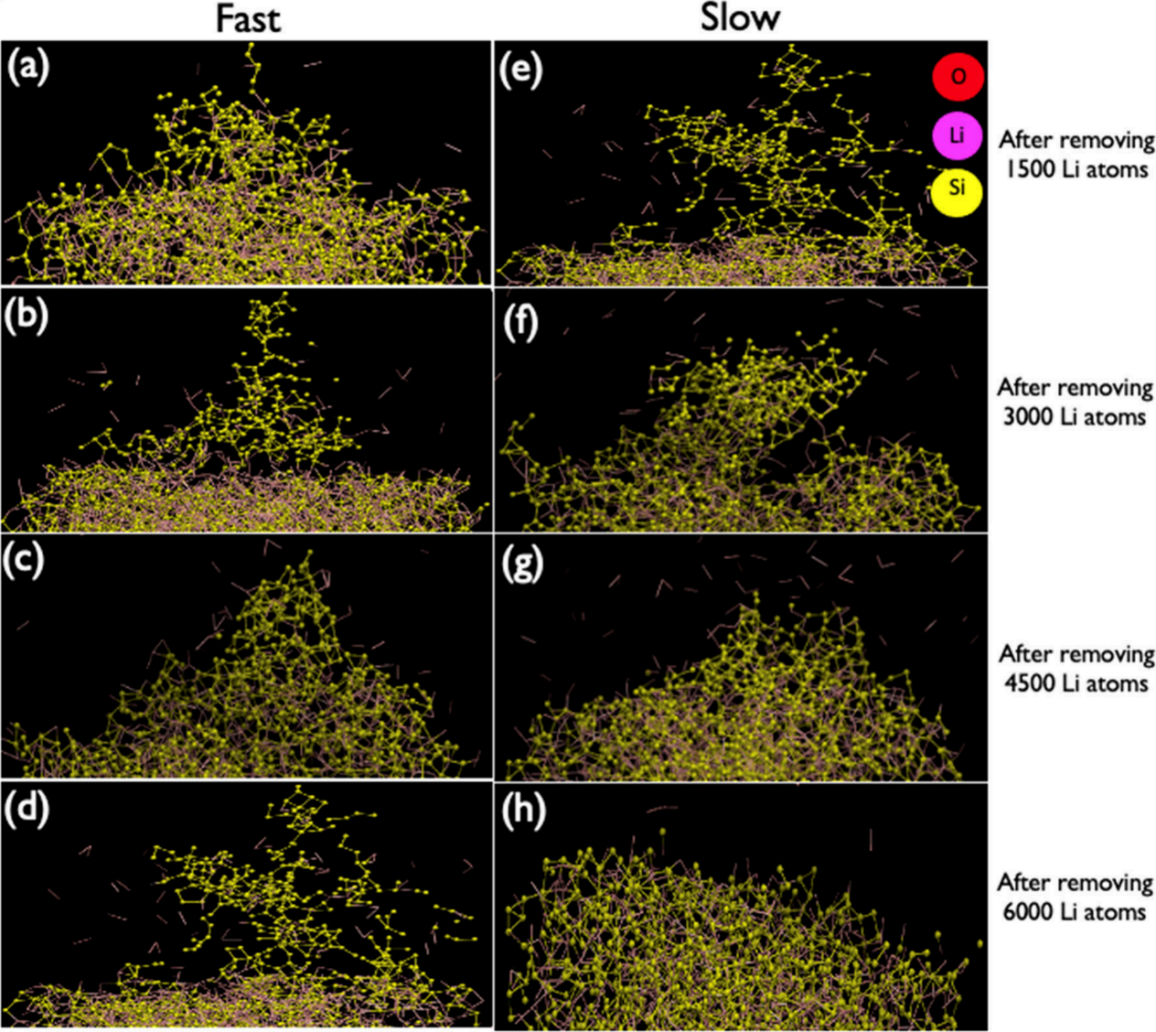
Illustration of Si dendrite formation when different delithiation conditions are applied to the initial a-Li^3.75^Si system. Two simulation snapshots visualizing the formation of dendritic shape structure during 1500, 3000, 4500, and 6000 Li atoms for (a–d) fast and (e–h) slow removal.

The radial distribution functions (RDF) analysis (Figure S10) highlights distinct structural differences between fast and slow delithiation for Si–Si, Li–Li, and Si–Li interactions. For Si–Si bonds, slow delithiation exhibits a sharp peak at ∼2.5 Å, reflecting tightly packed Si clusters, while fast delithiation shifts the peak to ∼2.7 Å and introduces secondary features (∼4 Å), indicating porous and scattered Si networks. Li–Li interactions show similar trends, with slow delithiation producing a sharp peak at ∼3 Å, signifying localized clustering, whereas fast delithiation broadens the peak (∼3.2 Å) and adds a secondary peak (∼4.8 Å), reflecting Li dispersion and inhomogeneity. Si–Li interactions under slow delithiation yield a stable peak at ∼2.7 Å with limited secondary features, while fast delithiation shifts the peak to ∼3.1 Å and intensifies secondary peaks (∼4.5 Å), signifying disrupted bonding and increased porosity. Overall, these findings confirm that slow delithiation preserves structural integrity, yielding denser and more uniform clusters, while fast delithiation induces significant disruption, contributing to porous structures and irregular surfaces. These trends align with our ReaxFF simulations and emphasize the importance of controlling delithiation rates to mitigate dendrite formation and improve the long-term performance of Si anodes. The formation of Si dendrites during delithiation is a result of both kinetic and thermodynamic factors. Kinetically, Li-ion removal destabilizes Li–Si interactions, increasing Si atom mobility and driving the formation of Si–Si bonds. These bonds form unevenly due to the inhomogeneous removal of Li, resulting in localized clustering of Si atoms. Faster delithiation rates exacerbate these effects, as the system lacks sufficient time to reorganize, leading to porous and irregular structures. Thermodynamically, the formation of Si–Si bonds is driven by the reduction of system free energy, as bond formation eliminates dangling bonds and minimizes surface energy. However, the inhomogeneous nature of Li extraction creates localized energy gradients that promote dendritic growth. At slow delithiation rates, the system can achieve more favorable energy states, forming denser and more uniform Si clusters. Additionally, entropy changes associated with Li removal and Si migration further contribute to the inhomogeneous atomic rearrangements that characterize dendritic structures. This interplay between kinetic and thermodynamic factors underscores the complexity of Si dendrite formation during delithiation.

The density of Li-ions around the Si atoms is reduced during Li-ion removal, and Si–Si bonds and clusters are formed inhomogeneously, depending on the local environment. The accompanied increase in surface area again facilitates further SEI growth and an increase of the electrode thickness and resistance over time. Also, the generation of highly inhomogeneous surfaces will lead to large differences in localized Li-ions extraction rates, promoting a further increase in Si dendrite formation.

The unfavorable implications of the Si dendrite formation on the lifetime of Si anodes suggest that lifetime can be improved significantly if proper mitigation methods against dendrite formation can be found. This includes employing a strategy to control the local delithiation rate, limiting the depth of lithiation, or either encapsulating or alloying the active material to prevent further dendritic growth.

Contrary to the prevailing notion that Si degradation primarily arises from particle fracturing during cycling, our findings reveal a more-complex degradation pathway for Si as an acitve material in LIBs. We observed a transformation of the Si surface into a dendrite-like rendering particles with an extremely high active surface area. Based on molecular dynamics simulations and electron microscopy investigations of cycled electrodes, we attribute the formation of the observed surface morphology to an inhomogeneous reorganization of the Si atoms during delithiation. TEM micrographs show that this causes an extremely high growth of SEI, reduced porosity, and increased electrode thickness, as well as a large amount of Li trapped in the SEI layers. This increases the resistance and reduces the amount of Li available for cycling, further driving the degradation mechanisms.

## Supporting Information Available

Experimental section, TEM sample preparation and details on computational methods (including schematics), additional TEM images as a function of cycles (PDF)
