# Supplementary material for: Revisiting Mechanism of Silicon Degradation in Li-Ion Batteries: Effect of Delithiation Examined by Microscopy Combined with ReaxFF
Source: J Phys Chem Lett. 2025 Feb 21;16(9):2238–44. doi: 10.1021/acs.jpclett.4c03620 (PMC11891961; doi:10.1021/acs.jpclett.4c03620)
Supplement: Supplementary file 1 — jz4c03620_si_001.pdf [file jz4c03620_si_001.pdf]

Revisiting mechanism of Si degradation in Li-ion batteries: effect of delithiation examined by microscopy combined with ReaxFF.

Carl Erik L. Foss[1*], Mahdi K. Talkhoncheh[2], Asbjørn Ulvestad[1], Hanne F. Andersen[1], Per Erik Vullum[5], Nils Peter Wagner[5], Kenneth Friestad[4], Alexey Y. Koposov[1,3*], Adri van Duin[2] & Jan Petter Mæhlen[1*]

[1] Department of Battery Technology, Institute for Energy Technology
P.O. Box 40, NO-2027 Kjeller, Norway
[2] Department of Chemical Engineering, Pennsylvania State University, University Park, Pennsylvania 16802, United States
[3]Centre for Material Science and Nanotechnology, Department of Chemistry, University of Oslo, P.O. Box 1033, Blindern, 0371 Oslo, Norway
[4] Elkem, PO Box 8040 Vaagsbygd, NO-4675 Kristiansand, Norway
[5] SINTEF Industry, P.O. Box 4760, NO-7465 Trondheim, Norway

Supporting Information

Experimental Methods

General.

The following materials were used as received for the fabrication of electrodes and cells without any further chemical modifications: Si particles (eSi-400, Elkem), conductive additive (C65, IMERYS), graphite (KS6L, IMERYS), carboxymethyl cellulose (CMC, MW = 90 000, Sigma-Aldrich), KOH, deionised H_2O (18 MΩ), Cu-foil (Schleck), 2032-type coin-cells (Hohsen), electrolyte - 1M lithium hexafluorophosphate in (1:1:3 v/v/v) ethylene carbonate: propylene carbonate: dimethyl carbonate + 1%wt vinylene carbonate + 5%wt fluoroethylene carbonate (Solvionic), membrane (Celgard). The galvanostatic measurements of the cells were performed using a battery tester from Arbin instruments.

Electrode preparation.

Si particles were ball-milled with 30 ZrO (Ø10 mm) balls for 5 min at 800 rpm in a planetary micro mill (Fritsch P7 Premium) with a ball-to-mass ratio of 8.5. 10g (56.22 wt %) of the ball-milled Si powder was mixed with 2.5g (14.05 wt %) conductive additive, 1.66g (9.67 wt%) graphite, and 2.5 g (14.05 wt%) carboxymethyl cellulose as a binder. In addition, 1.12 g (6.3 wt%) of buffer solution (pH=3), prepared with citric acid, potassium hydroxide and deionised H_2O (2.5:1 solvent to solid ratio), was added. The resulting slurry was mixed for 40 min at 2000 rpm (Thinky mixer), then deposited on dendritic Cu-foil using screen printing technique: 200 mesh, wire

thickness of 40 μm and emulsion thickness of 25 μm - 200/40/25. The electrodes were then dried in a vacuum oven for 3 hours at 120°C.

Cell fabrication.

The Si-based electrodes were evaluated in a half-cell configuration using Li foil as a counter electrode in a climate controlled chamber at 25 °C. 16 mm diameter electrode discs with loading of 0.72 ±0.04 mg/cm^2 were cut and then moved into an Ar-filled glovebox (MBRAUN) where the cells were assembled using 2032-type coin-cells (Hohsen). A total amount of 50 μL electrolyte was added, and a microporous polypropylene monolayer membrane (20 μm thick) was used as a separator. The following protocol was used for the cells cycled at full capacity: two formation cycles at C/20 (179 mA/g_{Si}) followed by cycles at C/5 (716 mA/g_{Si}) with upper voltage limit set to 1.0V *vs* Li/Li^+ and lower voltage limit set to 50 mV *vs* Li/Li^+. The Si-based electrodes were also evaluated in limited capacity cycling, where the lithiation cut-off was set to 1200 mAh/g_{Si}. A nominal capacity of 3121 mAh per gram of active material, calculated assuming 3579 mAh/g for Si and 372 mAh/g for graphite, was used in finding the Equivalent Full Cycles (EFC; calculated total accumulated capacity divided by nominal capacity) and the State of Health (SoH; 100 x capacity over nominal capacity).

FIB-SEM/TEM

The transmission electron microscopy (TEM) lamellae were made by focused ion beam (FIB) preparation using a Helios G4 UX from Thermo Fisher Scientific. Carbon-based protection layers were first deposited on top of the region of interest. The first part of the protection layer mas made by e-beam assisted deposition to avoid any ion-beam damage and implantation into the top surface

of the electrode. The lamellae were cut out and transferred to a Cu TEM grid before final thinning. All coarse thinning was done with 30 kV acceleration voltage for the Ga+ ions. Final thinning was first done at 5 kV and then at 2 kV on either side of the lamella to minimize surface damage.

TEM was performed with a double spherical aberration corrected, cold field emission gun JEOL ARM 200FC, operated at 200 kV. Energy dispersive x-ray spectroscopy (EDS) and dual energy electron energy loss spectroscopy (EELS) were performed simultaneously in scanning transmission electron microscopy (STEM) mode. A large solid angle Centurio detector (100 mm2 detector covering a solid angle of 0.98 sr) was used for EDS and a GIF Quantum ER was used for EELS.

SEM characterisation was also performed with the FIB-SEM . For SEM/TEM analysis, the cells were stopped after a predetermined number of cycles (2, 20, 67, 78 and 200) to investigate the morphology at different stages. The cells were disassembled inside an Ar-filled glovebox and rinsed with diethylene carbonate (Sigma Aldrich) prior to analysis.

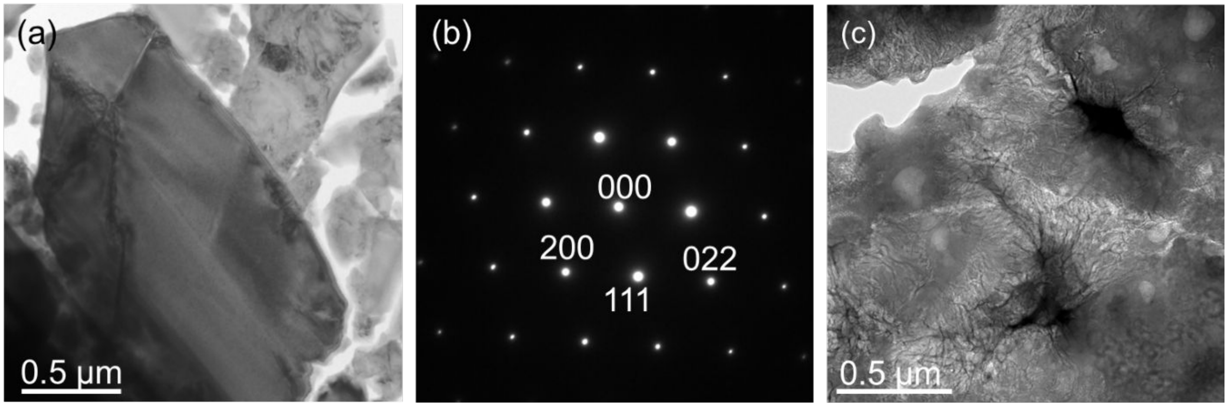

Figure S1. *(a) Bright field TEM image and (b) corresponding electron diffraction pattern of a Si particle prior to any cycling. (c) Bright field TEM image of two Si particles after 78 cycles showing only minor solid bodies, but with a lot of dendrites. Prior to cycling, the Si particles are single crystals, as demonstrated in the diffraction pattern in (b). In the image in (c), Si is significantly heavier than the SEI phases and therefore gives dark contrast in the image. A network of thin, dendritic Si is sticking out from the remaining solid body of the Si particles.*

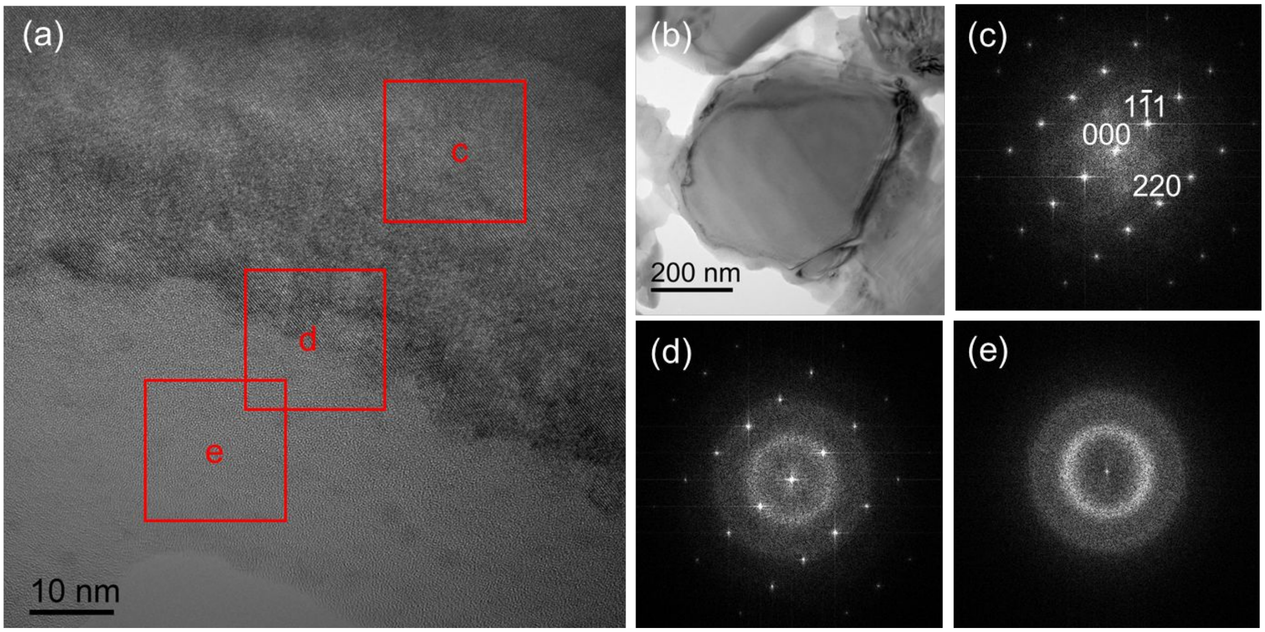

Figure S2. *(a) High resolution, bright field TEM image from a surface-near region of the Si particle shown in the bright field image in (b). The Fourier transforms in (c) – (e) are from the red framed regions in (a) and demonstrate the transition from a crystalline Si core to an amorphous Si shell. The amorphous halos from the amorphous region have d-spacings that correspond with what is expected from amorphous Si.*

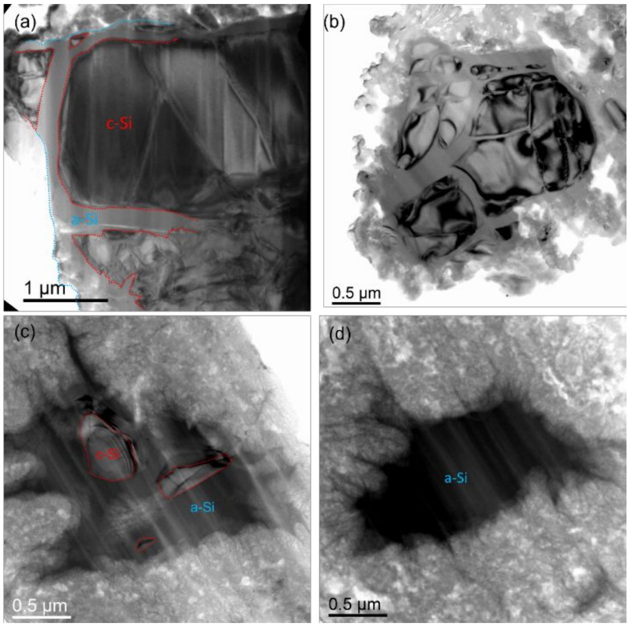

Figure S3. Bright field TEM images of typical Si particles after (a) 2 formation cycles, (b) 20 cycles, (c) 80 cycles, and (d) 100 cycles. All cells were cycled with limited capacity at 1200 mAh/g_{Si}. Red lines mark the interface between amorphous and crystalline Si.

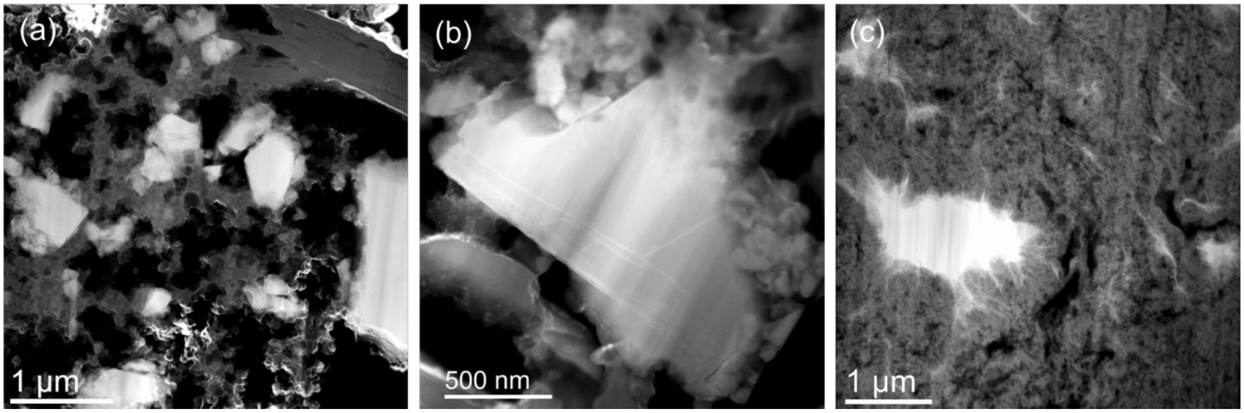

Figure S4. HAADF STEM images of electrodes after (a) 2, (b) 20, and (c) 100 cycles.

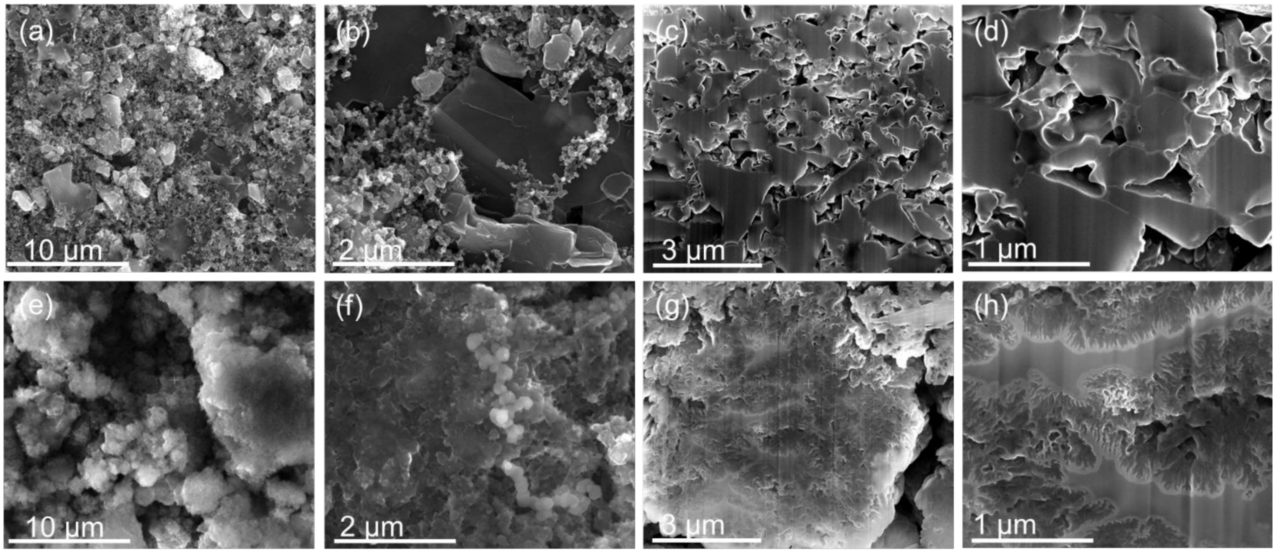

Figure S5. (a-b) Surface and (c-d) cross-section SEM images of an uncycled electrode. On the row below similar (e-f) surface and (g-h) cross-section images are shown from an electrode cycled 78 times. Prior to cycling, the Si particles have a smooth surface. After 78 cycles, much of the porosity is filled with varios SEI phases and the Si particles have totally changed their morphology.

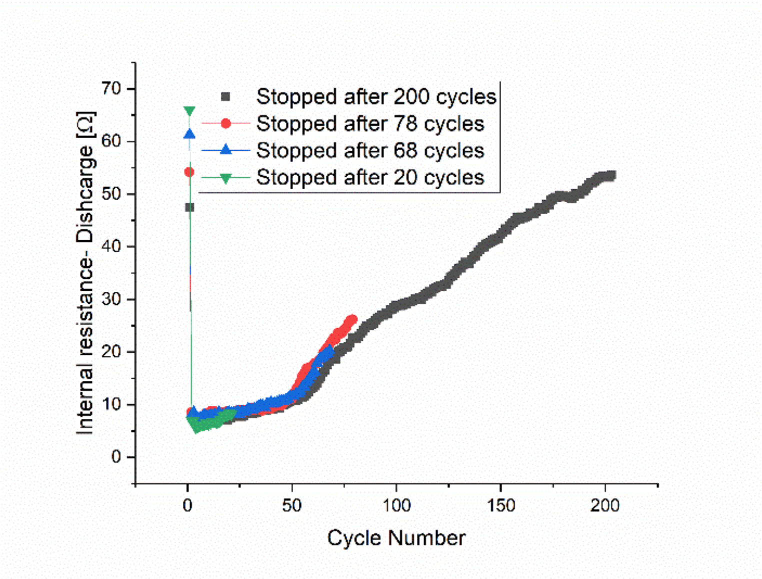

Figure S6. Internal resistance increases during discharge as a function of cycle.

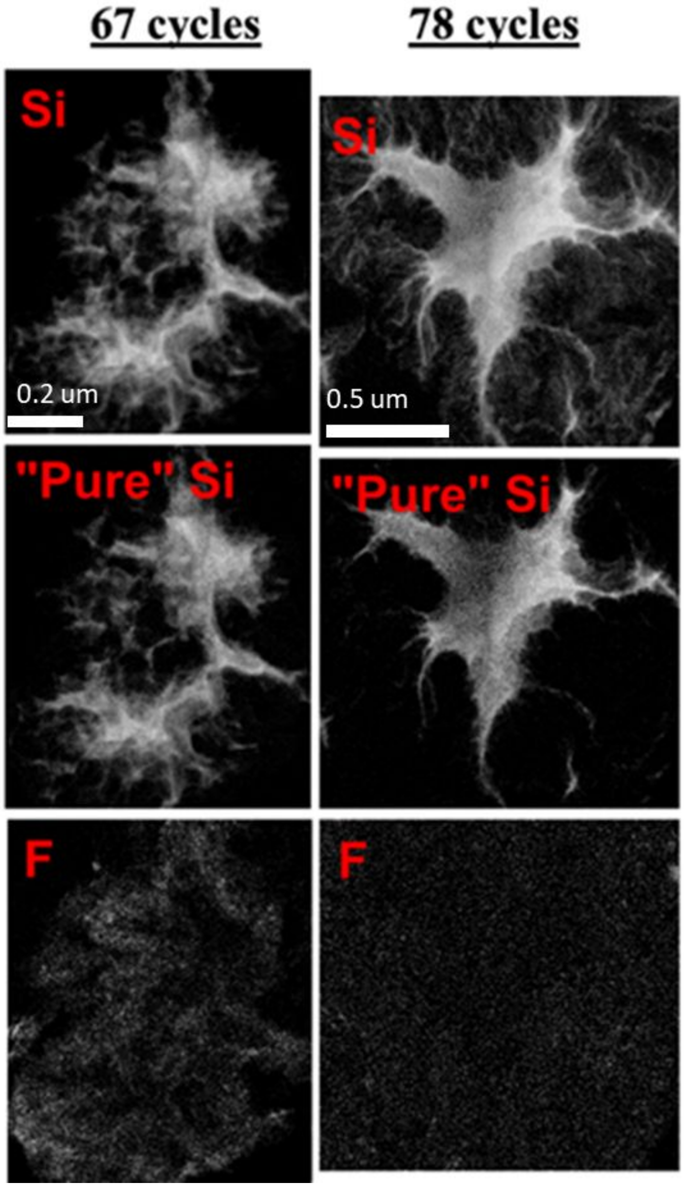

*Figure S7***.** *The Si map shows all present Si, independent of the oxidation state of Si. The "Pure" Si map shows only the Si that has an oxidation state lower than 2. This means SiO_x where $0 \leq x < 1$. The amount of oxidized Si is higher after 78 cycles compared to 67 cycles.*

Computational methods

In this study, we employed the Amsterdam Modeling Suite (AMS) combined with the Reactive Force Field (ReaxFF) potential to perform reactive molecular dynamics simulations of silicon-lithium interactions. Specifically, we utilized the Li/Si/O force field parameters developed by Ostadhosssein et al.[i] to model the degradation processes occurring during lithiation and delithiation. The simulation workflow is detailed in Figure S.8.

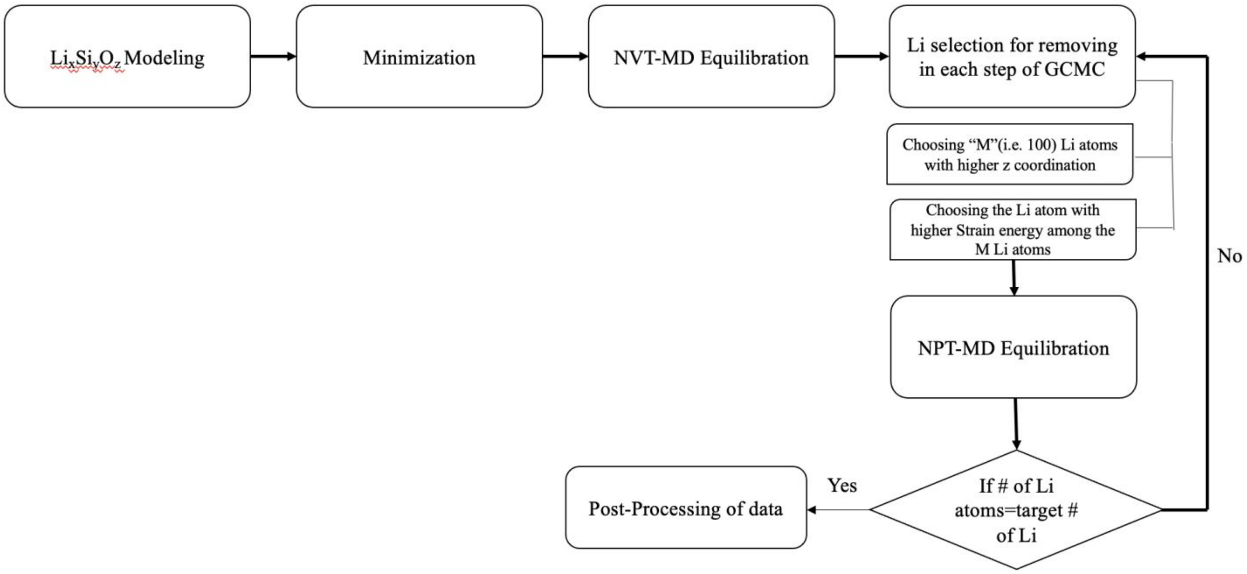

Figure S8. Continuous delithiation algorithm: Combined GCMC and reactive molecular dynamics simulation (Reaxff) algorithm. GCMC method is utilized to remove Li GCMC method is utilized to remove Li atoms from lithiated Structure after minimization and NVT equilibration. In each step of GCMC, Li atoms with higher z-coordination and strain energy is removed and system is equilibrated under NPT ensemble simulation

The initial geometries were constructed by creating Li_4Si and Li_4SiO_x (where x accounts for less than 5% of Li atoms) blocks on the surface of a crystalline silicon (c-Si) system, oriented in the z-direction, with a total of more than 5000 Li atoms. These geometries were relaxed using the conjugate gradient minimization scheme to eliminate any high-energy configurations. Following relaxation, lithiation and delithiation processes were simulated using a combination of the Grand Canonical Monte Carlo (GCMC) method and reactive molecular dynamics simulations. This

approach allowed us to dynamically study the interactions between lithium and silicon atoms during the charge and discharge cycles. The GCMC simulations were conducted using an in-house Python-based code, which enabled us to control the Li insertion and removal steps effectively. The simulation protocol began with an NVT equilibration at 50 K for 10 ps to eliminate any hot spots in the system. This was followed by a gradual temperature increase (T-regime) from 50 K to the target temperature (700 K) at a rate of 2.5 K/ps. Lithiation and delithiation were then performed in steps of 2000 fs under NPT conditions, as illustrated in Figure S.9. The use of the GCMC method combined with ReaxFF simulations allowed us to capture the dynamic interactions and structural transformations of the silicon anode under varying lithiation and delithiation conditions.

The temperature of 700 K was chosen for the molecular dynamics simulations to accelerate atomic diffusion and facilitate the observation of key lithiation and delithiation mechanisms within computationally feasible timescales. This approach is a widely accepted practice in the field, as elevated temperatures enable the simulation of rare events such as Si-Si bond breakage, atomic migration, and clustering, which occur over much longer timescales at experimental conditions. Moreover, 700 K is well below the melting temperature of silicon (1687 K) and the eutectic temperature of Li-Si alloys, ensuring that the system remains in the solid state. While the chosen temperature exceeds typical operating conditions, it allows us to probe the fundamental atomic-scale processes that govern the structural and chemical evolution of Si anodes during cycling. Importantly, the qualitative trends observed at 700 K align with experimental findings, demonstrating the relevance of the results to real-world applications

To investigate the dendritic shape formations during battery degradation, and structural changes of a-$Li_{3.75}SiO_x$ thin film upon delithiation align with electrochemical measurements and

TEM/SEM results, a continuous delithiation algorithm was developed (Figure S8). This novel systematic delithiation algorithm, helps to capture the effect of different delithiation rate, which plays an important role in the irreversible structural change during the delithiation of Si. Besides, the fundamentals of degradation were investigated by analysing the relationship between the depth of discharge and corresponding volume and structural changes at different rates.

We performed an equilibration of the box in three stages (as shown on Figure S9a): the first stage is performed at 50 K for 10 ps to eliminate any hot spots in the initial geometry. Next, the temperature is increased from 50 K to target temperatures (700K) at a rate of 2.5 Kps^{-1} in NVT (constant volume, temperature) ensemble. Finally, in the third stage, the box is equilibrated at the target temperature for 2000 fs after removing each Li atom by using NPT equilibration. Temperature and pressure were regulated using the Berendsen thermostat and barostat. Periodic boundary conditions for x and y direction and reflective wall conditions for z-direction were employed in all three directions, and a time step of 0.25 fs was used for all the simulations in this study. At equilibrium state, the chemical potential of all Li atoms should be the same in the surface and inside the Si cluster. Decreasing Li concentration in the surface layer will decrease the Li chemical potential led to migration of Li atoms out of Si cluster. This Li chemical potential gradient will naturally drive Li atoms to diffuse out of a-$Li_{3.75}Si$ film in order to reach equilibrium. On the basis of this concept, a step-by-step delithiation algorithm was applied. A fixed number of Li atoms, ΔN= 100 in this paper, was randomly removed at each delithiation step from the lithiated Si cluster from the top surface. Then, the structure was subject to NPT MD simulations for relaxation (with the Berendsen thermostat and the velocity Verlet integration algorithm at a time step of 0.1 fs) at 700 K. A temperature of 700 K is far from the melting temperature of Si (1687 K).

The usage of 700 K in this study only accelerates the diffusion and decreasing the simulation time without changing the diffusion mechanism. This can be explained as both experiments[ii] and molecular dynamics simulations (based on DFT or ReaxFF)[iii] have obtained diffusivity for Li−Si system at high temperatures ranging from 600 to ∼1500 K then extrapolated the room- temperature diffusivity using the Arrhenius equation. The extrapolated room temperature Li diffusivity from the ReaxFF- MD simulation agreed well with experimental results. The relaxation time (Δt) will allow the Li inside the a-Li_xSi film to diffuse to the surface layer. To emulate the "natural" out-diffusion of Li atoms of Si cluster at different delithiation rates (ΔN/Δt), Δt was used as a regulation factor. Delithiation stages were continued until an insufficient amount of Li atoms remained in the a-Si cluster to be removed, which can be considered as a "fully delithiated state". A delithiation rate of 50 Li/1 ps is equivalent to a current density of 3.2×10^7 A/cm^2, which is much higher than the typical current densities used in experiments (10^{-3}∼10^{-6} A/cm^2).[iv] The fast delithiation rate in MD simulation is expected and similar to the high strain rate used in MD simulations of deformation processes.[iv] Δt for the a-LixSi structure to reach equilibrium at each delithiation step is determined in a way the concentration gradient (Δc = 3%), caused by removing Li atoms stays fixed. The novel developed continuous delithiation algorithm was implemented to track the atomistic formation of dendritic shape structures on the surface of Li_xSi_y cluster subject to fast, and slow delithiation rates (Figure 4). At starting time of simulation t = 0, $Li_{3.75}Si$ structures were fully lithiated (Figure S9b). During each delithiation step, Li diffused out from the a-Li_xSi cluster to the amorphous surface layers in response to the chemical potential gradient induced by Li-removal from the surface, causing the surface layers to be refilled by Li atoms. Due to Li atoms continuous diffusion out of the a-Li_xSi, a significant volume contraction happened which is accompanied by formation of inner pores inside the cluster. The migration of Li atoms leads to

force Si atoms for migration toward the surface and forming dendritic shape structure on the surface layer as well as surface delamination.

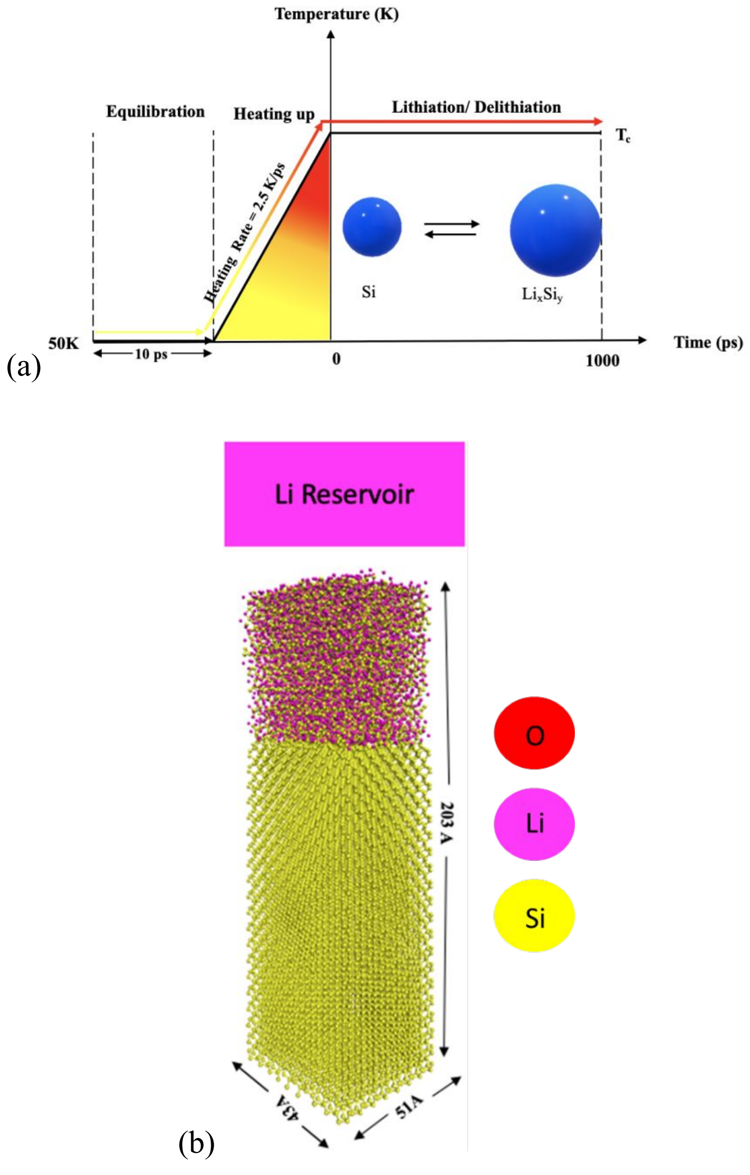

Figure S9. (a) Schematic representation of thermal history for all considered samples, (b) snapshots of the initial configurations for $Li_{3.75}Si$ after 10 ps of equilibration simulations at NVT ensemble with T = 700 K.

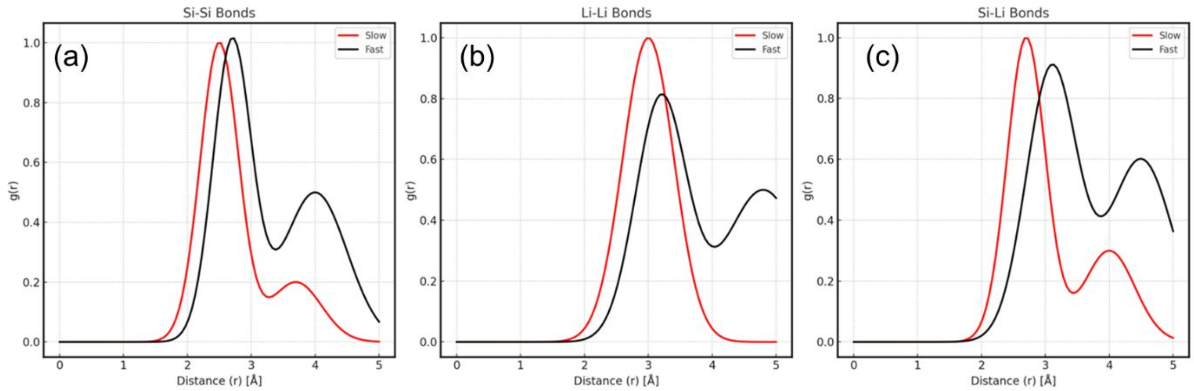

Figure S10. Radial distribution functions (RDFs) for (a) Si-Si, (b) Li-Li, and (c) Si-Li bonds under fast and slow delithiation conditions

---

[i] Phys. Chem. Chem. Phys., 2015, 17 (5), 3832-3840
[ii] J. Phys. Chem. A, (2016), 120, 13-25
[iii] J Phys. Chem. Chem. Phys. (2015), 8 ,81-90
[iv] Phys. Chem. Chem. Phys., 2015,17, 11301-11312
